# Ethnobotanical survey and antimycobacterial activities of plants used against tuberculosis in Lubumbashi, DR Congo

**DOI:** 10.1186/s41182-025-00745-1

**Published:** 2025-05-03

**Authors:** Evodie Numbi Wa Ilunga, Marsi Mbayo Kitambala, Kalunga Muya, Olivier Lachenaud, Joachim Mukekwa Maloba, Jean-Baptiste Lumbu Simbi, Véronique Fontaine

**Affiliations:** 1 Department of Pharmacology, Faculty of Pharmaceutical Sciences, University of Lubumbashi (UNILU), Lubumbashi, Democratic Republic of the Congo; 2 Unit of Microbiology, Bioorganic and Macromolecular Chemistry, Faculty of Pharmacy, Université libre de Bruxelles (ULB), Brussels, Belgium; 3 Department of Chemistry and Industries, Faculty of Sciences and Technology, University of Lubumbashi (UNILU), Lubumbashi, Democratic Republic of the Congo; 4 Meise Botanic Garden, Meise, Belgium

**Keywords:** Mycobacterium, Ethnobotany, Traditional medicine, Medicinal Plants

## Abstract

**Background:**

Tuberculosis is still a serious threat to public health in Africa and especially in the Democratic Republic of Congo, which is one of the eight countries with approximately two-thirds of the global cases of tuberculosis. Given the difficulties in accessing health care services and antitubercular treatments, indigenous population also uses plant-based traditional medicine. This study aimed to identify plants with antituberculosis potential in traditional Katangese medicine.

**Methods:**

Interviews were conducted on traditional healers using snowball sampling method. Ethnobotanical data were assessed by determination of the informant consensus factor and the relative frequency of citation. Guided field walks allowed to collect plants. Methanolic extracts were tested on *Mycobacterium smegmatis* and *Mycobacterium bovis* BCG using microdilution, diffusion and agar proportion methods. The cytotoxicity of the best extracts was evaluated by cell viability assay on the human cervical squamous carcinoma SiHa cell line. The 50% inhibitory concentration and minimal inhibitory concentration (MIC) were used to determine the selectivity index.

**Results:**

Thirty-eight plant species from 23 families were identified, most of which were from Fabaceae (16%). Eleven out of 17 plant extracts inhibited the growth of *M. smegmatis* at MIC ranging from 13 to 250 μg/mL. The methanolic extracts of *Zanthoxylum chalybeum* and *Parinari curatellifolia* showed MIC_99_ of 62.5 and 62.5–125 μg/mL, respectively, on *M. bovis* BCG and showed IC_50_ values of 28 and 20 μg/mL, respectively suggesting a low selectivity index. This study was the first to investigate the antimycobacterial activity of *Terminalia mollis*, *Phyllanthus muellerianus*, *Ochna afzelii*, and *Rothmannia engleriana.*

**Conclusions:**

The demonstration of antimycobacterial activity in the plants used in Lubumbashi against tuberculosis opens opportunities for more in-depth research into their chemical composition and toxicity, ultimately aiming to enhance their safety for treatment of tuberculosis.

**Supplementary Information:**

The online version contains supplementary material available at 10.1186/s41182-025-00745-1.

## Background

Human tuberculosis (TB) is an infectious disease caused mainly by *Mycobacterium tuberculosis*, also called Koch's bacillus (BK). In 2024, tuberculosis was the deadliest infectious disease worldwide, with an incidence of 10.8 million tuberculous people and 1.25 million deaths [1]. Low- and middle-income countries were the most affected, accounting for 99% of new TB cases [1]. Human immunodeficiency virus (HIV) infection is an important risk factor for active tuberculosis, which also reduces the tuberculosis treatment success rate [1–3]. Indeed, long-term anti-TB treatment (minimal 6 months) with potential side effects from drug regimens tends to reduce treatment adherence, contributing to increased treatment resistance [4].

Eighty percent world population uses some form of traditional, complementary, and integrative medicine (T&CM), beside biomedicine. However, safety, and/or efficacy have been relatively scarcely described. WHO has repeatedly called to integrate T&CM into healthcare systems, among others to improve reproducible traditional medicine quality [5]. Many ethnobotanical surveys have reported the use of plants to treat a number of infections in traditional medicine, including urinary tract infections [6], sexually transmitted infections [7], female sterility [8–10], etc.

Owing to difficulties in anti-TB supplies, the population of Africa also uses plant-based traditional medicine [11]. Various studies have been conducted to identify effective plant-based remedies against tuberculosis [12, 13] even in Africa [14–18], but none have investigated plant-based remedies from the Democratic Republic of the Congo (DRC). Given that there is a high TB incidence in the DRC (estimated at 318 cases/100 000 people in 2021 and that the coverage rate of anti-TB treatment is approximately 70% [19]), it is important to verify the efficacy and safety of the DRC plant remedies used against tuberculosis. Furthermore, phytochemical analysis of identified plants could lead to the discovery of anti-TB compounds with new mechanisms of action which are urgently needed to target multidrug-resistant TB.

The aim of this research was to identify plants used by traditional healers against tuberculosis and other respiratory diseases in Lubumbashi (DRC) and its surroundings and to evaluate their antimycobacterial and cytotoxic activities.

## Methods

### Study area

**Fig. 1:**
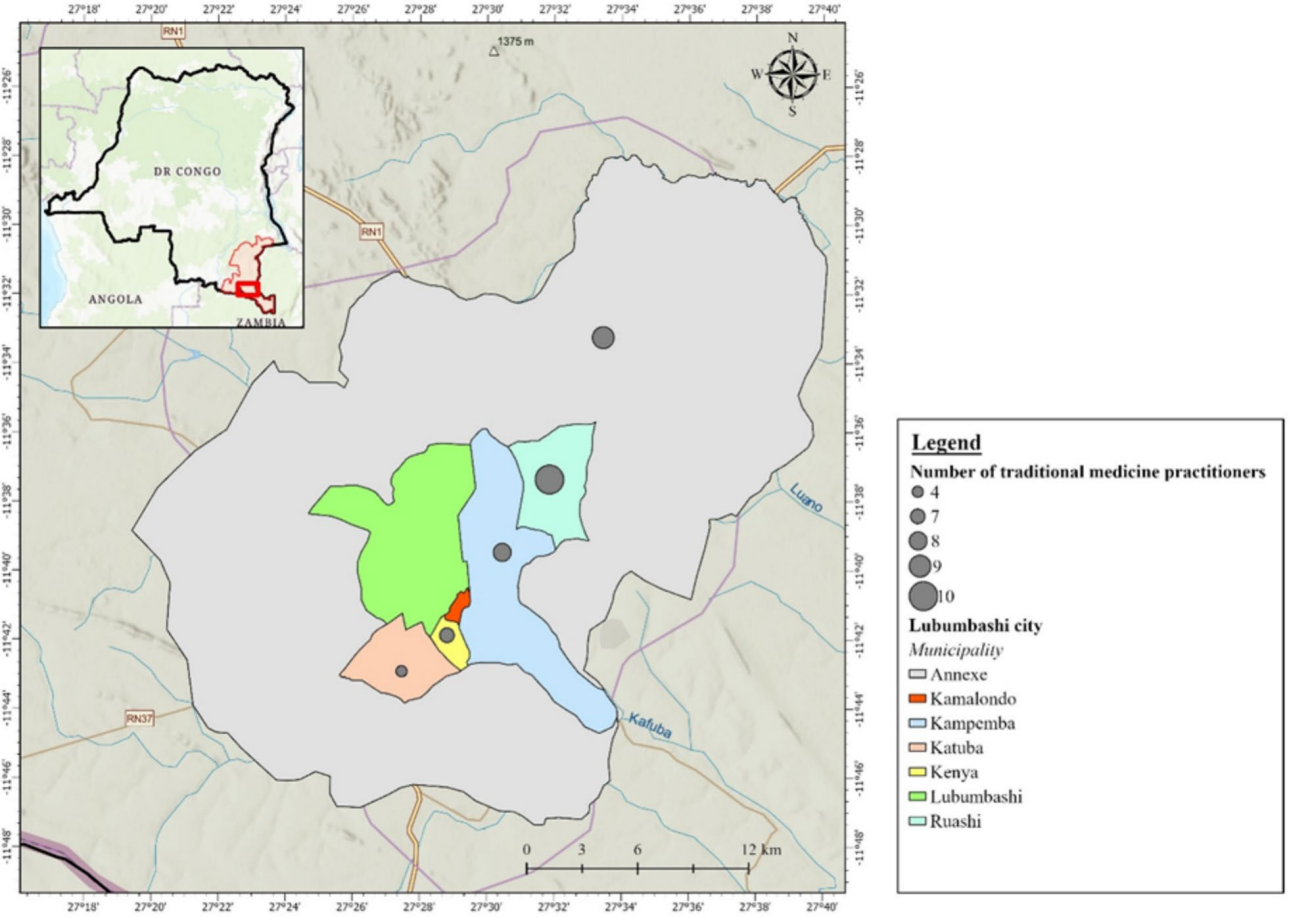
Geographical distribution of traditional medicine practitioners participating in the study

The ethnobotanical survey was conducted from January 5 to July 30, 2016, in five municipalities of Lubumbashi city, Kenya, Katuba, Ruashi, Annexe, and Kampemba (Fig. 1). Lubumbashi is the capital of the province of Haut-Katanga, located south of the DRC, at an altitude of 1230 m. The climate is tropical with two seasons (the dry season from April to November and the rainy season from November to March), an average annual rainfall of 1300 mm and an average temperature of 20 °C.

### Ethnobotany survey and plant collection

The medical ethic committee of the Université de Lubumbashi (CEM-UNILU) approved the study protocol (UNILU/CEM/025/2025).

As up-to-date directory of addresses of traditional medicine practitioners for the city of Lubumbashi was not available, a snowball sampling technique, as described by Cochran [20], was used to contact practitioners. The survey was conducted by direct interviews with preestablished questionnaire focusing on the interviewee’s socio-demographic characteristics (age, gender, tribe, acquisition mode of healing art, profession) and ethnobotanical information (plant name, other pathologies treated, part of the used plant, preparation method, administration route) for tuberculosis and other respiratory pathology management [21].

The plants were collected in the presence and with the guidance of traditional healers and botanical experts.

### Plant identification

First, plant species identification was performed by a highly skilled ethnobotanist, during plant collection, using scientific literature as reference. A first line identity verification was performed by RDC botanists by comparison between collected plants and samples kept at the INERA Kipopo Herbarium (DRC) but also using scientific literature. In Belgium, a second line of experts verified the identification of 17 plant samples obtained in DRC by comparison with scientific literature and samples kept in the Meise Botanical Garden Herbarium (Belgium).

### Preparation of extracts

The plants were dried in the shade, away from sunlight and humidity, and finely ground. To extract the maximum amount of the compounds, 50 g of plant powder were macerated in 500 mL of methanol p.a. (Fisher, purity 99.9%) for 24 h with stirring at room temperature and then rinsed once with the same volume. These extracts were filtered (Whatman No. 1 filter) dried on a rotary evaporator at a temperature ≤ 40 °C, and then stored at 4 °C. To compare the antimycobacterial activity obtained under our experimental conditions with that of traditional practitioners, the plants with the best activity were subjected to aqueous extraction via the decoction of 20 g of plant powder in 200 ml of Milli-Q water. The extract obtained was treated as previously described [22, 23].

### Extracts and reference product preparation

Stock solutions of the plant extracts were prepared in dimethyl sulfoxide (DMSO) at a concentration of 10 mg/mL and sterilised via syringe filter diam. 25 mm, pore size 0.2 μm. Stock solutions of rifampicin (5 μg/mL) and 1 mg/mL of ofloxacin were prepared in Milli-Q water, and 10 mg/mL orlistat was prepared in DMSO.

### Antimicrobial susceptibility assays

The sensitivity test of mycobacteria to plant extracts was carried out in two phases. All the plant extracts were first tested on *M. smegmatis* LMG 08190 (BCCM, Ghent, Belgium). Only extracts showing activity against this strain were tested on the *M. bovis* BCG GL2 Pasteur strain (CIP, Paris, France).

*Mycobacterium smegmatis* was maintained on tryptic soy agar (TSA) and incubated at 37 °C for 48 h. The *M. smegmatis* inoculum was prepared in sterile NaCl solution (0.9%, w/v) from a TSA preculture to obtain a suspension comparable to the 0.5 McFarland turbidity standard. Disk diffusion assays were performed as previously described [24]. Ten microliters (equivalent to 50 mg of methanolic extract) were loaded on sterile blank disks (Difco, Detroit, USA) placed on Muller Hinton agar. After 24 h of incubation at 37 °C, growth inhibition zones (diameters) were measured.

The minimum inhibitory concentration (MIC) was determined via the microdilution method as described previously [23], with some modifications. Serial twofold dilutions of each extract and reference product were carried out in 96-well plates with concentrations ranging from 250 µg/mL to 1.9 µg/mL for extracts and from 25 µg/mL to 0.09 µg/mL for ofloxacin and 0.1 µg./mL to 0.001 µg/mL rifampicin; then, each well was inoculated with 100 μL of bacterial suspension.

The MIC values were first interpreted after 48 h of incubation at 37 °C, corresponding to the lowest extract concentrations able to visually inhibit bacterial growth. This visual interpretation was verified by the addition of triazolyl blue tetrazolium bromide (98%; Acros Organics) to reach 0.5 mg/mL final concentration. This allowed visual detection of viable cells through formazan crystal formation, after 3 h of incubation at 37 °C. Culture medium was used as negative control (without bacteria), dimethyl sulfoxide was used as positive control and rifampicin was used as a positive drug control in the microdilution and proportion agar assays on *M. bovis* BCG. Ofloxacin was used as a positive drug control in the disk diffusion assay and microdilution methods on *M. smegmatis*. The bactericidal activity of the antimicrobial extracts and drugs against *M. smegmatis* was visually assessed by spreading, on a TSA plate, 10 µL well culture of a 96-well plate microdilution assay. The extract or drug concentration resulting in at least 2 log survival reductions, based on colony forming unit (CFU) counts after 48 h of incubation, was determined as the minimal bactericidal concentration (CMB).

*Mycobacterium bovis* BCG precultures were maintained in Middlebrook 7H9 broth (DIFCO) supplemented with 10% (v/v) Albumin Dextrose Catalase (Becton Dickinson), 0.05% (v/v) Tween 80 (Sigma‒Aldrich) at 37 °C. To assess the antimycobacterial activity of *M. bovis* BCG, microdilution and agar proportion methods were used. The microdilution method was performed in 7H9 medium supplemented with 10% ADC and 0.2% glycerol, as previously described [23]. The agar proportion method was performed in 7H11 medium supplemented with 10% OADC (oleic acid, bovine albumin, dextrose, sodium chloride, and catalase supplement) and 0.5% (v/v) glycerol (Sigma‒Aldrich), as previously described [25]. A total of 9.5 g of 7H11 culture medium was dissolved in 450 ml of Milli-Q water containing 2.5 mL of glycerol, and then 10.8 ml was distributed in different culture tubes with screws and autoclaved at 121 °C for 10 min. The mixture was cooled to ≤ 50 °C in a water bath. Then, 1.2 mL of OADC and the desired dilution of extract solution or rifampicin were added to each tube, mixed well while shaking gently, and the contents were poured into Petri dish and left to solidify.

After the medium solidified, 20 µl of 3 × 10^6^ CFU/mL inoculum were spread on each Petri dish until the inoculum was completely absorbed onto the medium containing samples to be tested. Dilutions of inoculums 3 × 10^5^ and 3 × 10^4^ CFU/mL were sprayed onto media without plant samples as growth controls. All the plates were incubated at 37 °C. The results were determined by comparing the number of colonies obtained on test plates, with tested extracts or reference antibiotics, with growth controls [26].

The MIC_99_ represents the lowest drug concentration that inhibited visible bacterial clump formation when growth of the 1% inoculum drug-free control became visible. The MIC_50_ values were visually determined based on the observation of an approximate 50% growth reduction.

### Cytotoxicity assay

The cytotoxicity assays of the best extracts were carried out on the human cervical squamous cell carcinoma SiHa cell line via the MTT cell viability test, as previously described [27]. The assay was performed in 96-well plates. SiHa cells (10,000 cells in 100 µL/well) grown in Dulbecco’s modified Eagle’s medium (DMEM) supplemented with 10% (v/v) foetal bovine serum (FBS) were first seeded in a 96-well plate. After 24 h of incubation at 37 °C with 5% CO_2_, 100 µL twofold serial dilutions of plant extracts, DMSO (negative control), or tetrahydrolipstatin/orlistat (positive control) in 10% FBS/DMEM were added to 96-well plates. Plates were incubated at 37 °C in 5% CO_2_ for 48 h then, washed two times with 150 μL per well with phosphate-buffered saline (PBS) before  incubation 4 h at 37 °C in 5% CO_2_ with 0.5 mg/mL 3-(4,5-dimethylthiazol-2-yl)−2,5-diphenyltetrazolium bromide (MTT, 98% Sigma–Aldrich) in 10% FBS/DMEM. After 4h incubation, plates were washed two times with PBS, and formazan crystals were solubilised within 100 µL DMSO.

Absorbances at 570 nm (the maximum formazan absorbance wavelength) and 630 nm (the background noise wavelength) were recorded via a spectrophotometer (Synergy HT, Bio-Tek). The raw data were transferred to a Microsoft Excel sheet and analysed. The 50% inhibitory concentration (IC_50_) values were determined. The selectivity index (SI) was calculated (IC_50_/MIC_99_) [28]. An SI lower than or equal to 1 indicates that the tested extract had greater cytotoxic activity than antimycobacterial activity, reflecting a poor or unacceptable selectivity index.

### Data analysis

Ethnobotanical data were assessed using various indicators. Informant consensus factors (ICF) were calculated as proposed by Heinrich et *al*. [29] to assess informant agreements to treat pathology categories using a specific plant, using the formula: ICF = Nur-Nt/Nur-1; where Nur is the number of times a specific disease category is mentioned and Nt is the number of plants mentioned for the treatment of this disease category by all informants.

The relative frequency citation was calculated using the formula proposed by Tardío and Pardo-de-Santayana [30], RFC = FC/N, where frequency of citation (FC) is the number of informants mentioning the use of the plant species and N is the number of informants taking part in the survey.

Data were entered in Microsoft Excel 365 and descriptive statistics such as frequency, mean, percentages (%) and standard deviation were determined. Antimycobacterial activities data were entered in Microsoft Excel 365 and exported to R software version 4.4.2 for statistical analyses. Normality was tested to assess whether data followed parametric or non‐parametric distribution. Consequently, the Kruskal–Wallis test was used to compare the MIC of the methanolic extracts from different plants with positive control activity (reference antibiotic). A p value < 0.05 was considered statistically significant.

## Results

### Ethnobotanical survey results

The ethnobotanical survey was conducted among 47 resource persons from Lubumbashi and its surroundings. The interviewees were mostly men (87.2%), with 47.96 ± 9.36 years of age (the oldest was 63 years, and the youngest was 33 years old). All interviewees belonged to nine ethnic groups, with the Luba ethnic group being the most represented (44.7%), followed by the Hemba (19%), and the Bemba (17%) groups. Regarding the professions of the interviewed people, farmers were the most represented (25.5%), followed by traditional healers (23.4%). Most of the interviewees (87.3%) acquired the art of healing through intergenerational knowledge transfer in the family, and only 6.4% of the interviewees acquired it through personal research.

The survey allowed to record 47 plant species, among which thirty-eight plants were collected and identified. The investigation allowed us to identify plant vernacular names, used plant parts, plant recipes, methods of drug preparation, treated pathologies, and routes of administration (Table 1).

**Table 1:** Ethnobotanical information on recorded plant species and identification

Scientific Names [Family, Voucher N°]	Local names	Organ^a^	Use to treat^b^	Prep^c^	Mode of administration	Informer
*Acacia sieberiana* DC. var. woodii (Burtt Davy) Keay & Brenan [Fabaceae, 407–2623]	Mumunshia kibamda (luba)	SB	Cough	D	Drink	T26
*Aframomum alboviolaceum* (Ridley) K. Schum. [Zingiberaceae, 686–3204]	Matungulu pori (swahili)	LE	Cough	D	Drink	T12
*Afzelia quanzensis* Welw. [Fabaceae, 87–1376]	Mupapa (bemba)	RO	Diarrhea, hernia, pain, cholera, asthma	D	Drink	T5
*Albizia adianthifolia* (Schumach.) W.Wight. [Fabaceae, 410–763]	Kapeta nzonvu (luba), kapeta Nsofu (bemba), kiskyaze (rund), haenga luvula (hemba)	LE, RO	Cough, diarrhea, abdominal pain	D	Drink	T9, T1, T20, T34
*Annona reticulata* Linn. [Annonaceae, 31–4964]	Cœur de bœuf (français), Mustafere (swahili), Moebe (hemba)	LE	TB, amoebic dysentery, H	D, M	Drink	T14, T22
*Annona senegalensis* Pers. [Annonaceae, 31–2665]	Mulolo (luba)	LE	Cough, fever	Sm	Aerosol	T37, T3
*Antidesma venosum* E. Mey ex Tul. [Euphorbiaceae, 230–119]	Kifubye (luba), kifumbya (bemba)	LE, RO	TB, H, bronchitis diarrhea	D, M	Drink Enema	T23
*Baphia bequaertii* De Wild. [Fabaceae, 478–837]	Kapalepale(bemba), kapalankeke (luba)	RO	TB, malaria, Cancer, HT, TS	D, I	Drink Enema	T31
*Brachystegia boehmii* Taub. [Fabaceae, 76–3039]	Musamba (luba)	RO	Healing, cough	C	Suction	T3, 38
*Cissus schmitzii* Dewit [Vitaceae, 679–1490]	Lenda (luba)	RO	TB, SW, sterility, gastritis, dysentery	D, M	Drink	T16
*Crossopteryx febrifuga* (G.Don.) Benth. [Rubiaceae, 575–6186]	Kububa (luba), mutambe lungu (bemba)	LE	Bacterial infection, diarrhea, cough	D	Drink	T21
*Cymbopogon nardus* (L.) Rendle. [Poaceae, 271–5179]	Kikotshi (swahili)	SB	Fever, cough	D, I	Enema, Fumigation	T20
*Diplorhynchus condylocarpon* (Müll.Arg.) Pichon [Apocynaceae, 39–353]	Mwenge (lamba), Mubudi (luba), Humwenge (bemba)	RO SB	Cough, H mycosis TB, amoebic dysentery	D, I, M	Drink	T10, T13, T19, T36
*Entandrophragma delevoyi* De Wild. [Meliaceae, 398–2801]	Kamertileza, leza (tabwa)	RO	Cancer, typhoid fever, mycosis, TB, SW	D	Drink	T2, T3
*Ficus ovata* Vahl [Moraceae, 424–602]	Mutaba (bemba)	LE, SB	Cough	D	Drink	T12, T8
*Ficus sansibarica* Warb.[Moraceae, 423–139]	Tshuyu (hemba), chilemba (bemba)	LE, RB	Cough, persistent wounds	D	Drink	T33, T10
*Ficus stuhlmannii* Warb. [Moraceae, 425–797]	Mupulampaka (bemba)	LE, SB	Cough, anemia	D	Drink	T21
*Harungana madagascariensis* Lam. Ex Poir. [Hypericaceae, 326–1454]	Mukuta (luba), kafifi (bemba)	SB	Cough, TB, malaria, hernia, gonorrhea, icterus	D, M	Drink Enema	T4, T14
*Hexalobus monopetalus* (A.Rich.) Engl.& Diels. [Annonaceae, 32–847]	Nkoyo (luba)	LE	Genitourinary infection, hernia, SW, diabetes, cough, cyst	M	Drink	T38
*Mucuna poggei* Taub. [Fabaceae, 516–2480]	Nyoka luipeta (luba), pese (swahili et bemba), kafu kanyama, mbese(hemba)	SB	Malaria, TB, abortion, HT, urinary tract disease	D, M	Drink	T22, T37, T20
*Ochna afzelii* R.Br. ex Oliv. [Ochnaceae, 445–634]	Munyawe (bemba)	LE, SB	TB, malaria, typhoid fever, SW	D, M	Drink	T2
*Parinari curatellifolia* Planch.ex Benth. [Chrysobalanaceae, 567–5604]	Kikele Mutchi (Luba), kifulu mutshi	RO	Malaria, cough, TB, cancer	D	Drink	T18
*Pavetta schumanniana* F. Hoffm.ex K.Schum. [Pavettaceae, 594–1017]	Makawesha (bemba, lamba), kitumbotumbo (swahili)	LE	Cough, infection, TB	M	Drink	T20, T13, T7, T11
*Phyllanthus muellerianus* (Kuntze) Exell [Euphorbiaceae, 244–1443]	Lulembalemba (luba), mulembalemba (bemba), Alembalemba(hemba) musuganlanga, luanga ndindi (tshiluba)	LE, RO	Cough, sore, convulsion, H, HT, heartache	D, I	Drink	T26, T24
*Piliostigma thonningii* (Schumach.) Milne-Redh. [Caesalpiniaceae, 73–2447]	Kifumbe (luba), kifumbe (bemba), hifumbe (hemba)	RO, LE, SB	TB, HT, H, female sterility, diarrhea, cancer, abdominal pain, Cough, heartache	D	Drink	T15, T24, T27, T13
*Pseudolachnostylis maprouneifolia* Pax [Euphorbiaceae, 247–2656]	Kisembe (luba), musana (lala), musangati (bemba)	LE	Cough, gonorrhea	D	Drink	T24, T17
*Psorospermum febrifugum* Spach [Hypericaceae, 327–620]	Mukuta (luba), kafifi (tabwa)	LE, RO	Cough, abdomen pain, wound	D	Drink	T32
*Rauvolfia caffra* Sond. [Apocynaceae, 43–6047]	Mwimbe (bemba)	LE	Cough, heartache	D	Drink	T37
*Rothmannia engleriana* (K. Schum.) Keay [Rubiaceae, 600–604]	Mulualua (luba)	RO	Cough, H	D, I	Drink	T25, T22
*Sansevieria trifasciata* Prain [Liliaceae, 362–2029]	Mashiyangulungu (tshiluba), matwi a ngulungu (luba)	LE	Cough, pain	D	Drink	T28, T38
*Securidaca longepedunculata* Fresen.[Polygalaceae, 544–1660]	Lupapi (Kizela, Kibemba), Mweyeye (Swahili), Muchacha (tshokwe), Konse-konse (bemba	RO	TB, SI, gastritis, sterility, diarrhea, asthma, cough, cholera	M, D, I	Drink	T15, T20
*Solanum incanum* L. [Solanaceae, 640–1167]	Ntuntunya (luba, swahili), Umuntuntunya (bemba), Kalumba (tshokwe), kazirajani (Rund)	SB	H, panaris, tooth decay, wound, TB	D	Drink,	T20, T18
*Strychnos spinosa* Lam. [Longaniaceae, 370–77]	Lutonga (luba), nsanza ou zanga (bemba)	SB	TB, Sc, STD, typhoid, H malaria, flu, TS	D, M	Drink	T6, 30, T35
*Tephrosia vogelii* Hook.f. [Papillionaceae, 529–6165]	Bubake (songe), buba (luba)	LE	TB	M	Drink	T16
*Terminalia mollis* M.A.Lawson [Combretaceae, 126–136]	Kibobo (luba)	LE	Diarrhea, cough, and hernia	D	Drink	T11, T29, T4
*Xylopia katangensis* De Wild. [Annonaceae, 31–5764]	Muninambulu (bemba)	LE	Cough, stomach ache, diarrhea	M	Drink	T30
*Zanthoxylum chalybeum* Engl [Rutaceae, 616–5347]	Mpupwe kiulu (Luba), Pupwe (bemba)	RO	Sc, diabetes, cough, malaria	D	Drink	T29, T12
*Ziziphus mucronata* Willd. [Rhamnaceae, 662–558]	Nkakona (bemba), kankolenkole (lala)	LE	Cough, diarrhea, wound	D	Drink	T32

^a^SB: stem bark; RO: roots; LE: leaves

^b^TB: tuberculosis; SW: sexual weakness; H: hemorrhoid; HT: hypertension; Sc: schistosomiasis; STD: sexually transmitted disease; TS: testicle swelling; SI: sexual impotence

^c^D: decoction; M: maceration; I: infusion; Sm: smoke; C: calcination

The most used plant organs were fresh or dried leaves (44%), followed by roots (32.6%) and stems (22.4%) (Table 1). Decoction (60.4%) was the most used mode of preparation for traditional recipes. Maceration was the second recorded preparation method (24.5%). Crushing, squeezing or powdering were recorded non-systematically for decoction, maceration or infusion. Smoking was used by two informants for *Annona senegalensis*. Chewing was not reported. The oral route (80%) was the main route of administration. Salt was added to the drink when using *P. muellerianus* and* A. adianthifolia* and to the ash when using *Brachystegia boehmii* (Table 1). Four plants were the most cited: *Piliostigma thonningii* (Schumach.) Milne-Redh, *Pavetta Schamunniana* F. Hoffm. ex K. Schum., *Diplorhynchus condylocarpon* (Müll.Arg.) Pichon and *A. adianthifolia* (Schumach.) W. Wight, with an RFC of 0.11; followed by *Mucuna poggei*, *Strychnos spinosa,* and *T. mollis* (RFC = 0.08). Fourteen species were cited two times (RFC = 0.05) and 17 species were cited only once (RFC = 0.02) (Table 2).

**Table 2:** Relative frequency of citation

Plant species	FC	RFC
*Albizia adianthifolia*	4	0.11
*Diplorhynchus condylocarpon*	4	0.11
*Pavetta schumanniana*	4	0.11
*Piliostigma thonningii*	4	0.11
*Mucuna poggei*	3	0.08
*Strychnos spinosa*	3	0.08
*Terminalia mollis*	3	0.08
*Annona reticulata*	2	0.05
*Annona senegalensis*	2	0.05
*Brachystegia boehmii*	2	0.05
*Entandrophragma delevoyi*	2	0.05
*Ficus ovata*	2	0.05
*Ficus sansibarica*	2	0.05
*Harungana madagascariensis*	2	0.05
*Phyllanthus muellerianus*	2	0.05
*Pseudolachnostylis maprouneifolia*	2	0.05
*Rothmannia engleriana*	2	0.05
*Sansevieria trifasciata*	2	0.05
*Securidaca longepedunculata*	2	0.05
*Solanum incanum*	2	0.05
*Zanthoxylum chalybeum*	2	0.05
The leftovers	1	0.02

FC: citation frequency; RFC: relative frequency of citation

Besides their use to address tuberculosis and other respiratory tract pathologies, these plants were also intended against several other pathologies (Table 3).

**Table 3:** Informant consensus factor (ICF) about disease categories

Disease category	Number of species	Disease citation	ICF
Respiratory tract diseases	38	47	0.20
Diseases of the digestive tract	18	32	0.45
Urogenital diseases	13	24	0.48
Parasitic diseases	6	7	0.17
Metabolic diseases	8	10	0.22
Other (fever, headache, pain, fungus, anemia, jaundice and convulsion, wound)	15	19	0.22

The informant consensus factor determination (Table 3) showed that informant mostly agreed about plants to treat urogenital diseases (ICF = 0.48). This agreement was less strong for the digestive tract diseases, metabolic, and other diseases. The discrepancy in their answers was stronger for the respiratory tract diseases and even more for the parasitic diseases.

These plants were grouped into 23 families, among which Fabaceae were the most represented (26%), followed by Annonaceae (17%) (Table 1).

### Antimycobacterial and cytotoxic activities of plant extracts

The antimycobacterial activity of 17 plant species, represented by 20 methanolic extracts, was investigated first on *M. smegmatis* (Table 4).

**Table 4:** Antibacterial activities on *M. smegmatis*

Plant species and controls	Used organ^#^	Disk diffusion assay*	Microdilution assay	
			MIC_99_ (µg/mL)	MBC (µg/mL)
*Acacia sieberiana* DC. var. *woodii* (Burtt Davy) Keay & Brenan	SB	+	170 ± 78	208 ± 65
*Ficus sansibarica Warb*	LE	–	–	–
*Ficus stulmannii*	LE	–	–	–
*Hexalobus monopetalus* (A.Rich.) Engl. & Diels	LE	+	250	> 250
*Mucuna poggei* Taub	SB	–	–	–
*Ochna afzelii R.Br. ex* Oliv	LE	+	179 ± 67	> 250
*Parinari curatellifolia Planch.ex Benth*	RO	+	54 ± 15	115 ± 26
*Pavetta schamanniana* F. Hoffm.ex K.Schum	LE	–	–	–
*Phyllanthus muellerianus (Kuntze) Exell*	RO	+	170 ± 78	250
*Piliostigma thonningii* (Schumach.) Milne-Redh	LE	–	–	–
	SB	+	161 ± 87	229 ± 51
	RO	–	–	–
*Psorospermum febrifugum Spach*	LE	–	–	–
*Rothmannia engleriana* (K. Schum.) Keay	RO	+	196 ± 67	> 250
*Securidaca longepedunculata* Fresen	RO	+	214 ± 61	> 250
*Strychnos spinosa* Lam	SB	+ +	196 ± 67	250
	LE	–	–	–
*Terminalia mollis* M.A.Lawson	LE	+	89 ± 33	143 ± 47
*Zanthoxylum chalybeum* Engl	RO	+	13 ± 4	31 ± 24
*Ziziphus mucronata* Wild	LE	–	–	–
Ofloxacin		+ + +	0.19	0.39

^*^ + : ≥ 9- < 11 mm inhibition zone; +  + : ≥ 11 to < 13 mm inhibition zone; +  + + : ≥ 13 mm inhibition zone; -: no activity

^#^SB: stem bark; RO: roots; LE: leaves

Only 11 extracts, corresponding to 11 plant species, presented a minimum inhibitory concentration (MIC) less than or equal to 250 μg/mL on *M. smegmatis* (Table 4). Three most active methanolic extracts on *M. smegmatis* were extracts from *Z. chalybeum* (MIC = 13 µg/mL), *P. curatellifolia* (MIC = 54 µg/mL), and *T. mollis* (MIC = 89 µg/mL) (Table 4) The Kruskal–Wallis test showed an overall significant difference (p = 0). However, Dunnett's post hoc test revealed that the three extracts didn’t differ significantly from each other in their antimycobacterial activity against *M. smegmatis* (*p* > 0.05), although their activities were significantly higher (*p* < 0.05) compared to the other active extracts (Table 4). Furthermore, seven methanolic extracts showed bactericidal activity on this bacterial species (Table 4). The 11 extracts with antibacterial effects on *M. smegmatis* were further tested on *M. bovis* BCG (Table 5).

**Table 5:** Antimycobacterial activities toward *M. bovis* BCG GL2 and cytotoxicity of extracts

Plant species	*M. bovis* BCG					SiHa	
		Microdilution		Agar proportion			
	Organ^#^	MIC_50_^*^	MIC_99_^*^	MIC_50_^*^	MIC_99_^*^	IC_50_^*^	SI
**Methanolic extracts**							
*Acacia sieberiana* DC. var. woodii	SB		208 ± 72	250	–	ND	ND
*Hexalobus monopetalus* (A.Rich.) Engl.& Diels	LE	–	–	–	–	ND	ND
*Ochna afzelii* R.Br. ex Oliv	LE		208 ± 72	–	–	ND	ND
*Parinari curatellifolia* Planch.ex Benth	RO		62.5		125	20	0.16
*Phyllanthus muellerianus* (Kuntze) Exell	RO		250	–	–	ND	ND
*Piliostigma thonningii* (Schumach.) Milne-Redh	SB	–	–	–	–	ND	ND
*Rothmannia engleriana* (K. Schum.) Keay	RO	250	–	–	–	ND	ND
*Securidaca longepedunculata* Fresen	RO	–	–	–	–	ND	ND
*Strychnos spinosa* Lam	SB	–	–	–	–	ND	ND
*Terminalia mollis* M.A.Lawson	LE	–	–	–	–	ND	ND
*Zanthoxylum chalybeum* Engl	RO		62.5		62.5	28	0.45
**Aqueous extracts**							
*Parinari curatellifolia* Planch.ex Benth	RO		–	250	ND	95	0.38
*Zanthoxylum chalybeum* Engl	RO		–	> 250	ND	34	ND
**Controls**							
Orlistat						133	
Rifampicine		ND	0.01		< 0.06		

^#^SB: stem bark; RO: roots; LE: leaves

^*^Concentration in μg/mL

**Fig. 2:**
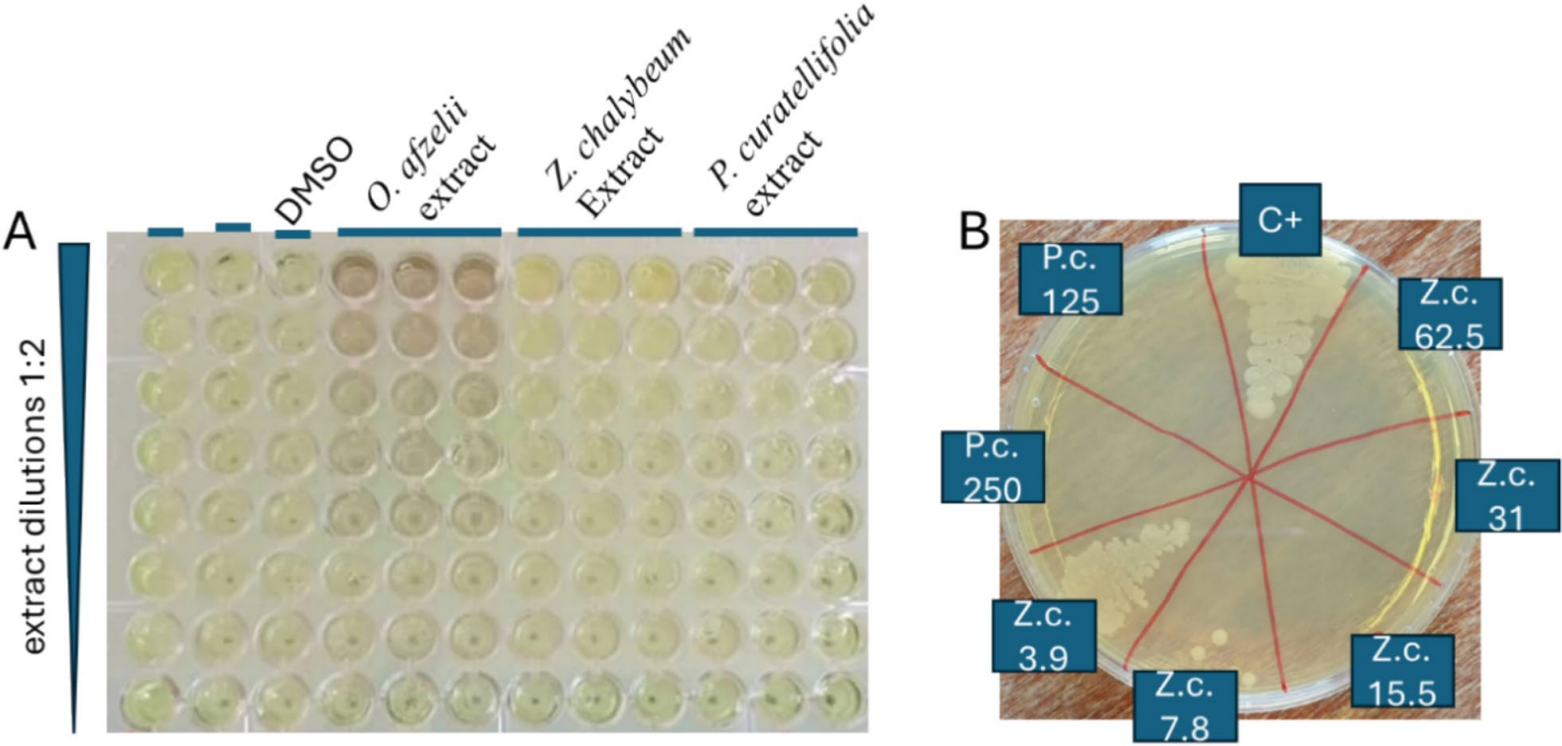
Antimycobacterial activity of *Ochna afzelii*, *Zanthoxylum chalybeum* (Z.c.), *and Parinari curatellifolia* (P.c.) methanolic extracts in the microdilution assay on *M. bovis* BCG (**A**), and on *M. smegmatis* for bactericidal activity (**B**). Numbers are extract concentrations in μg/mL

Six methanolic extracts inhibited the growth of *M. bovis* BCG. Five plant extracts exhibited activity with MICs ranging from 62.5 to 250 µg/mL, while *R. engleriana* showed weak activity with an MIC₅₀ of 250 µg/mL. The Kruskal–Wallis test indicated a significant difference overall (*p* = 0), however, the Dunnett post hoc test revealed no statistically significant differences between the MICs of the five plant extracts (*p* > 0.05). (Table 5, Fig. 2).

The methanolic extracts of the two most active plants (*P. curatellifolia* Planch.ex Benth and *Z. chalybeum* Engl) also showed cytotoxicity to the human cervical squamous carcinoma SiHa cell line. The IC_50_ and selectivity index (SI) were evaluated and were 20 μg/mL and 0.16, respectively, for *P. curatellifolia* and 28 μg/mL and 0.45, respectively, for *Z. chalybeum.* We also studied the antimycobacterial activities of aqueous extracts from the same organs of these two plant species (Table 3). Compared with the methanolic extracts, the aqueous extracts had lower or undetected antimycobacterial activities (MIC = 250 μg/mL for the *P. curatellifolia* aqueous extract and MIC > 250 μg/mL for the *Z. chalybeum* aqueous extract). However, the aqueous extracts also showed lower cytotoxicity (IC_50_ = 95 μg/mL and 34 μg/mL for the *P. curatellifolia* and *Z. chalybeum* aqueous extracts, respectively). Consequently, the *P. curatellifolia* aqueous extract showed an improved selective index (0.38), with twofold increase compared to SI of the methanolic extract (0.16).

## Discussion

The ethnobotanical surveys conducted in Lubumbashi showed that most traditional healers are adult men who acquire the art of healing through intergenerational knowledge transfer in the family. The same results were previously reported in the DRC [21, 31–34]. This can be explained by the fact that, in the African conception, those entitled to intergenerational healing knowledge transfer are foremost boys, future heads of the family, since the art of healing constitutes a manner of income.

Among the 47 plants listed by the traditional healers to treat TB in our survey, 38 were harvested and identified. Fabaceae were the most represented, as observed in previous ethnobotanical surveys conducted in the DRC and other African countries when investigating medicinal plants used to treat various pathologies [17, 18, 21, 22]. *Albizia adianthifolia* from the Fabaceae was one of the most cited plants*.* This is in agreement with the ethnobotanical study of Oryema *et al*., which recorded that 6.8% informants used the same plant in rural Uganda to manage symptoms of tuberculosis [35]. Another Fabaceae species, *P. thonningii*, which was also among the most frequently cited plants in our study, was previously reported to treat various conditions, including cough, tuberculosis, and diarrhea [36, 37]. The methanolic extract of the stem barks of this plant was only able to inhibit non-pathogenic *M. smegmatis.*

The plants documented in our ethnobotanical study are widely used to treat various pathologies. A low level of consensus among informants was observed in our study, with ICF values below 0.5 for all categories of pathologies. This may be partly attributed to the unique snowball sampling method employed in this study [38]. As highlighted by Espinosa *et al*. [39], this non-probabilistic approach does not ensure representativeness of general population. Moreover, an ICF value below 0.5 may indicate that a greater number of plant species could be used to treat the same condition, which could reflect the diversity of cultural and ethnic healing practices [30]. In addition, low ICF values may also suggest uncertainty among informants regarding the effectiveness of the reported medicinal plants. This may further explain the very low relative frequency of citation (RFC) values observed for the documented species.

To treat tuberculosis and other respiratory diseases with traditional medicine, leaves are mostly used (44.9%), and decoction is the most used preparation method (60.4%). Leaf collection is indeed easy to perform and allows preservation of plant species. Decoctions have the benefit of rapid release of the active ingredient but also have the drawback of altering the heat-labile active compounds, unlike other preparation methods. Several administration routes can be used both in biomedicine and in traditional medicine (oral, anal, inhalation, and local application). As previously observed, the route mostly used by the interviewees was the oral route. It is also the most convenient to perform [40].

Eleven methanol extracts from *Acacia sieberiana, Hexalobus monopetalus, O. afzelii, P. curatellifolia, P. muellerianus, P. thonningii, R. engleriana, Securidaca longepedunculata, Strychnos spinosa, T. mollis, and Z. chalybeum* had antimycobacterial activity against *M. smegmatis* (MIC_99_ values between 13 and 250 μg/mL). The two most active extracts on *M. smegmatis*, from the stem roots of *Z. chalybeum* and *P. curatellifolia*, (MIC_99_ values of 13 ± 4 and 54 ± 15 µg/mg, respectively) were the most active extracts to inhibit *M. bovis* BCG, with MIC_99_ of approximately 62.5 μg/mL, depending on the susceptibility assay method. The antimycobacterial activity of *Piliostigma thonningii*, *Securidaca longepedunculata,* and *Hexalobus monopetalus* were previously described in similar studies with plants from South Africa [41–43], sharing with Lubumbashi the Miombo forest.

The antimycobacterial activity of the *Zanthoxylum chalybeum* plant was previously reported on saprophytic mycobacteria, *M. madagascariense* and *M. indicus pranii*. [44]. The MIC values were very high for both species (1.25 and 2.5 mg/mL, respectively) using methanolic and dichloromethane extracts of *Zanthoxylum chalybeum* stem bark. Nevertheless, the methanolic extracts are showing cytotoxic activity on SiHa cells. This was unexpected as the methanolic extract of this plant was previously reported to be non-toxic in a brine shrimp model [44].

According to previously published results, it is tempting to speculate on the potential role of some specific molecules in antimycobacterial extracts. Among them, a benzophenanthridine alkaloid, skimmianine, and sesamine, as well as the triterpenoid lupeol, have been isolated from *Z. chalybeum* root bark and shown antimycobacterial activity against *M. tuberculosis* H37Rv [45–47]. Other compounds, such as chelerythrine (IC_50_ = 3.616 ± 0.51 μM) and lupeol, have been shown to be cytotoxic [45, 48]. Although these studies have identified active compounds extracted with organic solvents, further studies should be performed to assess whether aqueous preparations performed at home, based on the advice of traditional healers, contain the same cytotoxic or antimycobacterial compounds.

The antimycobacterial activity observed with the methanolic extract of *Parinari curatellifolia* leaves on *M. smegmatis* agrees with the results obtained in other studies [49, 50]. Although the methanolic extract was cytotoxic, the aqueous extract was relatively weakly cytotoxic, in agreement with the results of Gororo [51]. Several studies have shown that β-sitosterol is responsible for antimycobacterial activity [52, 53]. Two cytotoxic diterpenoids, 13-methoxy-15-oxozoapatline and 13-hydroxy-15-oxozoapatline, were also identified in the *Parinari curatellifolia* root bark methanolic extract [54].

Importantly, in this study, screening assays were not performed in the presence of Tween 80 (0.05%). Indeed, this non-ionic detergent can affect the upper layer of the tuberculous mycobacterial cell wall membrane, resulting in a bias of antimycobacterial activity [55]. This could explain why some extracts have been previously reported to have antimycobacterial effects on tuberculous strains (including on the  H37Ra strain, with unrepresentative tuberculous cell wall), whereas these extracts were, under the present assay conditions, only active against the non-tuberculous *M. smegmatis* [23].

Notably, the present study was the first to investigate and to highlight the antimycobacterial activity of methanolic extracts of *T. mollis, P. muellerianus, O. afzelii, and R. engleriana*.

In this study, only the antimycobacterial and cytotoxic activities of two aqueous crude plant extracts from *P. curatellifolia* and *Z. chalybeum* were tested. In both cases, although the cytotoxicity was reduced, the aqueous extracts also had lower or absent antimycobacterial activity, reducing their interest in traditional medicine to treat tuberculosis but also to discover new anti-TB drug candidates. Nevertheless, the antimycobacterial activity of all aqueous plant extract recipes, which are composed of various plants and organs, was not investigated here. Nor did we assess the antimycobacterial activity of all the plants from the ethnobotanical survey.

## Conclusions

This is the first ethnobotanical study on plants used by traditional healers in the Lubumbashi area (DRC) to treat tuberculosis and other respiratory infections. An ethnobotanical survey conducted on 47 resource persons allowed the identification of 38 plants. Out of them, approximately 65% of 17 plant extracts showed antimycobacterial activity against *M. smegmatis* (nontuberculous mycobacteria). The methanolic extracts of two plants showed strong bactericidal activity against *M. smegmatis* and *M. bovis* BCG. However, these extracts were also cytotoxic to SiHa cells, unlike their aqueous extracts, which showed weaker or absent antimycobacterial activity. The antimycobacterial activities of *T. mollis*, *P. muellerianus*, *O. afzelii,* and *R. engleriana* were reported for the first time. More in-depth research should be carried out to identify the phytochemical compounds responsible for the antimycobacterial activity of these plants.

## Supplementary Information

Additional file 1

## Data Availability

Biological assay raw data that support the findings of this study have been deposited in Zenodo with the DOI: 10**.5281/zenodo.10213133.

## Abbreviations

ADC: Albumin dextrose catalase
ATCC: American type culture collection
BCG: Bacille de Calmette et Guérin
C: Calcination
CMB: Minimum bactericidal concentration
D: Decoction
DRC/DR Congo: Democratic Republic of Congo
H: Hemorrhoid
HT: Hypertension
ICF: Informant consensus factor
I: Infusion
INERA: Institut National pour l’Etude et la Recherche Agronomique
LE: Leaves
M: Maceration
MIC: Minimal inhibition concentration
OADC: Oleic acid, Albumin, Dextrose, Catalase
RFC: Relative frequency of citation
RO: Roots
Sc: Schistosomiasis
Sm: Smoke
SB: Stem bark
SI: Sexual impotence
STD: Sexually transmitted disease
SW: Sexual weakness
TB: Tuberculosis
TS: Testicle swelling
TS: Tryptone soy agar
UNILU: Université de Lubumbashi
ULB: Université libre de Bruxelles
