# Supplementary material for: Ethnobotanical survey and antimycobacterial activities of plants used against tuberculosis in Lubumbashi, DR Congo
Source: Trop Med Health. 2025 May 3;53:64. doi: 10.1186/s41182-025-00745-1 (PMC12049048; doi:10.1186/s41182-025-00745-1)
Supplement: Supplementary file 1 — Additional file 1 [file 41182_2025_745_MOESM1_ESM.docx]

**Supplementary Table 1. Family and voucher identity of the collected plant species**

|  | **Familly** | **Scientific name** | **Voucher specimen-album number** |  |
| --- | --- | --- | --- | --- |
| Annonaceae | | *Annona reticulata* Linn. | **31-4964** |  |
|  |  | *Annona senegalensis* Pers. | **31-2665** |  |
|  |  | *Hexalobus monopetalus* (A.Rich.) Engl.& Diels. | **32-847** |  |
|  |  | *Xylopia katangensis* De Wild. | **31-5764** |  |
| Apocynaceae | | *Diplorhynchus condylocarpon* (Müll.Arg.) Pichon | **39-353** |  |
|  |  | *Rauvolfia caffra* Sond. | **43-6047** |  |
| Caesalpiniaceae | | *Piliostigma thonningii* (Schumach.) Milne-Redh. | **73-2447** |  |
| Chrysobalanaceae | | *Parinari curatellifolia* Planch.ex Benth. | **567-5604** |  |
| Combretaceae | | *Terminalia mollis* M.A.Lawson | **126-136** |  |
| Euphorbiaceae | | *Antidesma venosum* E. Mey ex Tul. | **230-119** |  |
|  |  | *Phyllanthus muellerianus* (Kuntze) Exell | **244-1443** |  |
|  |  | *Pseudolachnostylis maprouneifolia* Pax | **247-2656** |  |
| Fabaceae | | *Acacia sieberiana* DC. var*. woodii* (Burtt Davy) Keay & Brenan | **407-2623** |  |
|  |  | *Afzelia quanzensis* Welw. | **87-1376** |  |
|  |  | *Albizia adianthifolia* (Schumach.) W.Wight. | **410-763** |  |
|  |  | *Baphia bequaertii* De Wild. | **478-837** |  |
|  |  | *Brachystegia boehmii* Taub. | **76-3039** |  |
|  |  | *Mucuna poggei* Taub. | **516-2480** |  |
| Hypericaceae | | *Harungana madagascariensis* Lam. ex Poir. | **326-1454** |  |
|  |  | *Psorospermum febrifugum* Spach | **327-620** |  |
| Liliaceae | | *Sansevieria trifasciata* Prain | **362-2029** |  |
| Longaniaceae | | *Strychnos spinosa* Lam. | **370-77** |  |
| Meliaceae | | *Entandrophragma delevoyi* De Wild. | **398-2801** |  |
| Moraceae | | *Ficus sansibarica* Warb*.* | **423-139** |  |
|  |  | *Ficus ovata* Vahl | **424-602** |  |
|  |  | *Ficus stuhlmannii* Warb. | **425-797** |  |
| Ochnaceae | | *Ochna afzelii* R.Br. ex Oliv. | **445-634** |  |
| Papillionaceae | | *Tephrosia vogelii* Hook.f. | **529-6165** |  |
| Pavettaceae | | *Pavetta schumanniana* F. Hoffm.ex K.Schum. | **594-1017** |  |
| Poaceae | | *Cymbopogon nardus* (L.) Rendle. | **271-5179** |  |
| Polygalaceae | | *Securidaca longepedunculata* Fresen. | **544-1660** |  |
| Rhamnaceae | | *Ziziphus mucronata* Wild*.* | **662-558** |  |
| Rubiaceae | | *Crossopteryx febrifuga* (G.Don.) Benth. | **575-6186** |  |
|  |  | *Rothmannia engleriana* (K. Schum.) Keay | **600-604** |  |
| Rutaceae | | *Zanthoxylum chalybeum* Engl | **616-5347** |  |
| Solanaceae | | *Solanum incanum* L. | **640-1167** |  |
| Vitaceae | | *Cissus schmitzii* Dewit | **679-1490** |  |
| Zingiberaceae | | *Aframomum alboviolaceum* (Ridl.) K. Schum. | **686-3204** |  |
